# Progress testing of an objective structured clinical examination during undergraduate clinical clerkship: a mixed-methods pilot study

**DOI:** 10.1186/s12909-023-04940-8

**Published:** 2023-12-14

**Authors:** Ikuo Shimizu, Junichiro Mori, Aiga Yamauchi, Sawako Kato, Yuichi Masuda, Yuichi Nakazawa, Hiroyuki Kanno

**Affiliations:** 1 Center for Medical Education and Clinical Training, Shinshu University School of Medicine, Matsumoto, Japan; 2 Department of Medical Education, Chiba University Graduate School of Medicine, Chiba, Japan; 3 Division of Safety Management, Chiba University Hospital, Chiba, Japan; 4 Academic Affairs Office, Shinshu University School of Medicine, Matsumoto, Japan; 5 Safety Management Office, Shinshu University Hospital, Matsumoto, Japan

**Keywords:** Clinical clerkship, Objective structured clinical examination, Progress testing, Rating scale, Self-directed learning

## Abstract

**Background:**

Progress testing is an assessment method in which an examination reflecting competencies at graduation is regularly administered to students over multiple years, thereby facilitating self-directed learning. However, the significance of the objective structured clinical examination as a progress test in undergraduate education, needs to be determined. This study provides evidence of the role of the objective structured clinical examination for progress testing and optimal scoring methods for assessing students in different academic years.

**Methods:**

We conducted a sequential explanatory mixed-methods pilot study. Participants were assessed using the Item Rating Scale, the year-adjusted Global Rating Scale, and the Training Level Rating Scale. The characteristics of each scale were compared quantitatively. In addition, the influence of the objective structured clinical examination as a progress test on learning attitudes was examined. Qualitative data from a post-examination questionnaire were analyzed, using content analysis to explore influences on self-directed learning.

**Results:**

Sixth and fifth year clinical students (*n* = 235) took the objective structured clinical examination progress test. The total Item Rating Scales were recorded (%) as 59.03 ± 5.27 and 52.64 ± 5.08 (*p* < 0.01); Training Level Rating Scale was 3.94 ± 0.39 vs 3.22 ± 0.42 (*p* < 0.01); and the year-adjusted Global Rating Scale was 4.25 ± 0.44 vs 4.32 ± 0.52 (no significant difference), for the sixth and fifth year students, respectively. The correlations across stations and the reliability of each station were satisfactory. Four categories were identified in the qualitative analysis: “motivation to learn during the clinical clerkship was promoted,” “dissatisfied with being asked about things they had not experienced,” “confusion about being unable to use conventional test-taking strategies,” and “insufficient understanding of competencies at graduation.” The scores indicated significant differences in performance according to training year.

**Conclusions:**

This study provides evidence that the objective structured clinical examination can be used as a progress testing tool for undergraduate clinical clerkships. Further enhancement of training opportunities and dissemination of performance competency goals in clerkship curricula are required if we intend to promote self-directed learning through progress testing.

**Supplementary Information:**

The online version contains supplementary material available at 10.1186/s12909-023-04940-8.

## Introduction

Progress testing (PT) is a testing method in which an examination at a level of difficulty that indicates the completion of learning (graduation) with a passing score, which is regularly given to students to help them understand their mastery of the expected achievement goals [1]. PT is cross-sectional in that it includes participants at diverse levels of training and allows for a longitudinal assessment through the opportunity to compare performance over time [2]. Owing to these characteristics, PT has been shown to have certain educational benefits in fostering self-directed learning (SDL) [3]. SDL is a learning attitude in which individuals take the initiative to diagnose their own learning needs, formulate their own learning goals, and identify resources for learning. They also choose the appropriate learning strategies and evaluate their learning outcomes, including performance in a national board examination [4, 5]. Ultimately, PT will curb shortsighted academic test preparation and cramming, promote deeper lifelong learning, and even improve performance on national certification examinations. However, most PTs that have been practiced to date are written examinations, although there are differences in the medium used, such as paper or computers [6]. Thus, PTs only provide information on the cognitive domain rather than emphasize demonstrating various competencies [7]. From an assessment theory perspective, using written examinations to assess the utilization of knowledge aspects often does not adequately measure them because it requires a reasonable amount of attention to the composition and other aspects of the test [8]. It is a weakness of PT as a written examination.

As direct observations of clinical skills are often insufficient in the workplace, the objective structured clinical examination (OSCE) is frequently used, in which learners are presented with simulated clinical problems to assess their clinical competencies in solving them [9, 10]. It is commonly used to assess various skills, such as history taking, physical examination, and clinical procedures, as well as other roles required by physicians, such as communication and professionalism.

However, the role of the OSCE as a PT (OSCE-PT) has yet to be fully explored. Few attempts reported on its implementation in postgraduate internal medicine training are by Pugh et al. [4, 5]. More advanced residents recorded higher scores and higher test reliability in their study [11]. They provided evidence for the validity of using scores achieved on the OSCE as a measure of progress for learners at different training levels, suggesting that the OSCE may play a role as a PT. In addition, it can also function as formative assessment, which is expected to have the potential to promote SDL [12]. However, the potential of PT in undergraduate education remains unclear. As a characteristic of undergraduate education, differences in performance across years in the OSCE-PT may be influenced by the attitudes of the target students toward learning. Compared with more passive learning methods such as classes, workplace-based learning requires SDL, which is also associated with lifelong learning attitudes. Therefore, if the OSCE can be inserted during the undergraduate clinical curriculum, where OSCE tasks of the same difficulty level are assigned to students in different years and the achievement level of the students’ skills can be assessed, it will help students understand the expected level of mastery of achievement goals and bring about SDL, which will also enable educators to provide more personalized skills education. Our research questions were as follows:

1. Does OSCE-PT measure improvement in competencies over time in an undergraduate medical education curriculum?
2. How does OSCE-PT influence SDL attitudes?

## Methods

In this study, we examined whether OSCE-PT could measure improvements in competencies over time in an undergraduate medical education curriculum, and how PT influenced SDL attitudes.

### Context of examination

**Fig. 1:**
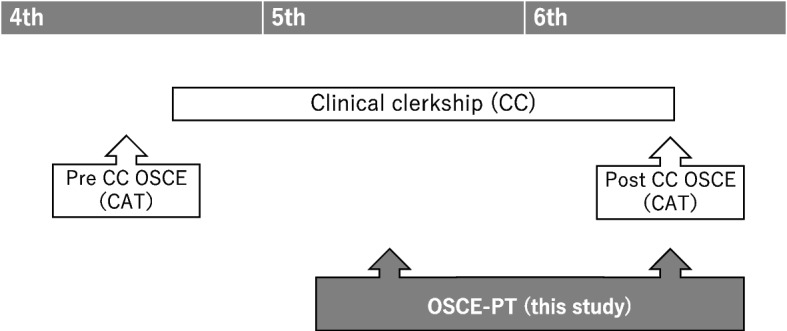
Overview of clinical skills assessment using the existing CAT-OSCEs in a typical medical school in Japan (Shinshu University) and the OSCE-PT in this study. Other assessment opportunities than these OSCEs were not described (e.g. workplace assessment, portfolio, written or oral tests) . In this study, OSCE-PT for the 6th year students was conducted simultaneously with post-CC OSCE. CAT, Common Achievement Test; CC, clinical clerkship; OSCE, objective structured clinical examination; PT, progress testing

In Japan, medical schools have a six-year undergraduate curriculum, with the clinical clerkship (CC) program dedicated to the last two years. Two types of OSCEs are implemented as summative assessment for clinical skills before and after clinical clerkship (Fig. 1). The former type of OSCE before starting clinical clerkship (“pre-CC”) is conducted as a part of the Common Achievement Tests (CAT). In addition, the latter type of OSCE to confirm whether the 13 tasks (Table 1) in the Model Core Curriculum [13] have been implemented after completing clinical clerkship (“post-CC”) as the CAT in 2020. Both OSCEs are summative assessments and cannot measure the achievement status of skills during clinical clerkship for feedback, as PT does. Other assessment opportunities, such as workplace-based assessments, are implemented for both formative and summative purposes, in addition to these OSCEs. These assessments were designed according to the blueprint for assessing clinical years (Table 2). Like other OSCEs, this blueprint is based on the clinical competencies shown in Table 1 [13]. Of these, we chose three stations (medical record, clinical procedures, and patient safety) for this examination because they have not been assessed in the existing OSCEs and are difficult to standardize in the workplace-based assessments.

**Table 1:** Tasks that should be performed at graduation as described in the Model Core Curriculum 2016 [13]. Thirteen tasks are defined as competencies at graduation

1. Listen to medical history and perform physical examination. 2. Think of differential diagnoses. 3. Interpret the results of basic laboratory tests. 4. Plan prescriptions. 5. Document medical records (medical chart). 6. Orally present the patient situation. 7. Clarify clinical problems and collect evidence. 8. Conduct/receive handover of a patient case. 9. Collaborate in an interprofessional team. 10. Perform initial responses to highly urgent patients. 11. Obtain informed consent. 12. Perform fundamental clinical procedures. 13. Contribute to patient safety through the identification and improvement of organizational issues.

**Table 2:** Blueprint for assessing clinical skills in a clinical clerkship curriculum of a typical medical school in Japan, and this study (OSCE-PT). This blueprint is created based on the example of Shinshu University, where assessment methods during CC vary for each rotation

Schedule	Assessment methods	Competencies at graduation (tasks should be performed) [13]												
		1	2	3	4	5	6	7	8	9	10	11	12	13
before CC (y4)	Pre-CC OSCE (CAT)	X									X		X	
during CC (y4–6)	workplace-based (including portfolio and oral tests)	X	X	X	X	X	X	X	X	X	X	X	X	X
end of CC (y6)	Post-CC OSCE (CAT)	X	X		X		X	X						
**midpoint (y5) and** **end (y6)** **of CC**	**this study** **(OSCE-PT)**			**X**		**X**			**X**	**X**		**X**	**X**	**X**

*CAT*, the Common Achievement Tests, *CC* clinical clerkship, *OSCE* objective structured clinical examination, *PT* progress testing

In addition to the existing OSCEs mentioned earlier, we implemented a “mid-term CC” OSCE in the fifth year, marking the midpoint of the clinical clerkship program. This assessment opportunity was utilized as an OSCE-PT (Fig. 1). As a pilot study exploring the role of the OSCE-PT, this study describes the function of OSCEs in assessing medical students across different years. Furthermore, the authors described the structure for recording scores and aimed to establish evidence for an appropriate rating scale for an undergraduate OSCE-PT.

The stations were developed by an educational specialist of the Center for Medical Education and Clinical Training who had received intensive training on simulation-based education and assessment through a consensus meeting of faculty. Medical interviews, physical examinations, clinical reasoning and planning, presentations, and discussions with health professions (i.e., the rest of the competencies) were not included in this study, because they were asked in existing OSCEs and regularly assessed in the workplace. The study stations took 20 minutes for charting medical records and 7 minutes for other stations. The purpose of the OSCE-PT was explained to the students in advance, and the results were provided after the examination.

### Participants

We conducted this study at Shinshu University School of Medicine, a national university in Japan. The pre-CC OSCE takes place at the end of the first semester of the fourth year. The clinical clerkship then begins in the second semester of the fourth year and continues until the first semester of the sixth year. The post-CC OSCE takes place just after the end of the clinical clerkship.

Two years of the six-year curriculum (from the second half of the fourth academic year to the first half of the sixth year) are devoted to clinical clerkships in this school. Thus, the study was conducted with students at the end of the first semester of the fifth year, when the clinical clerkship is in its intermediate stage, and with students taking the Post CC OSCE. We estimated that a sample of 126 students (63 each) would provide 80% power to detect a standardized effect size of 0.5 with a two-tailed type I error of 0.05 [14].

The OSCE-PT was administered to fifth and sixth year students who had participated in their CC program. Although testing was mandatory, participation in the study was voluntary, and written informed consent was obtained from those who wished to participate. This study was approved by the Institutional Review Board of Shinshu University School of Medicine (No. 4383).

### Assessment scales and rater

Three types of rating scales were used based on a previous study for assessing postgraduate residents [4], and their characteristics were compared; Item Rating Scale (IRS), Training Level Rating Scale (TLRS), and year-adjusted Global Rating Scale (yGRS). IRSs were developed to assess five specific requirements in each station. Content experts developed the original lists, and then educational experts reviewed and revised them by conducting iterative mock tests to identify potential defects beforehand and ensure the quality of stations. The items were rated on a six-point scale according to the following stages: preclinical (not allowed to start CC), just starting CC, during CC, completing CC (acceptable to graduate), during residency, and completing residency (can be entrusted to perform). TLRS was used to assess the overall performance of each station, and rated on a six-point scale like the IRS. The yGRS was used for the rating of examinee performance with a six-point scale to account for differences between years, with a score of four or higher considered acceptable for the examinees’ academic year (inferior, poor, borderline, acceptable, good, excellent).

There was one rater at each station. They regularly instructed medical students and residents, and were knowledgeable about their various performance levels. They also had experience with ratings of previous OSCEs, and had received prior instructions on the rating for each station. All performances were video recorded in case of any doubt regarding the ratings. Students from each year’s cohort were tested. This meant that the raters were able to ascertain the academic year of each examinee. However, the scores for other competencies during clinical practice were not communicated to the raters in advance.

### Analysis

Based on a pragmatic paradigm, we employed a sequential exploratory mixed methods research approach incorporating quantitative (OSCE assessment forms) and qualitative (examinees’ open-ended questionnaires) methods. In the quantitative analysis, t-tests were used to compare the results of the three types of assessment tables across academic years, and the IRS was used to determine the examinees’ score for each station, which was converted to a percentage score (%) after the total number of correctly answered items was added. For the TLRS and yGRS, the scoring values for each task were used. These data were used to calculate the mean scores for each task and the overall test. As a measure of effect size, we used Cohen’s *d*, where 0.20 corresponded to a small effect, 0.50 to a medium effect, and 0.80 to a large effect [15]. Correlations between measures were compared using Spearman’s rank correlation coefficient (*r*_*s*_), as the values of these measures were expected to be non-normally distributed, based on previous OSCE results. The internal consistency of each station was assessed using Cronbach’s alpha. Statistical analysis was performed using IBM’s SPSS version 27.0, with *p* < 0.05 considered significant.

In the qualitative analysis, the views of consenting students regarding the impact of this examination on their studies were elicited using an online open-ended anonymous questionnaire. Open-ended comments were analyzed using deductive content analysis, drawing upon Garrison’s conceptual framework for SDL [16] as the coding framework to define categories and sub-categories [17, 18]. In this framework, SDL is described as self-management, motivation, and self-monitoring. All authors read comments independently and were involved in the extraction of content; two authors (IS, JM) conducted open coding and discussed the coding. After verifying that the data across all comments possessed no excess or deficiencies in interpretation, we determined theoretical saturation. To ensure the reliability of the findings, the categories and subcategories were regularly reviewed by an author with extensive experience in qualitative research (IS) for content review. All authors agreed to the final results. We referred the Good Reporting of A Mixed Methods Study framework to secure the quality of the research [19].

## Results

The OSCE was conducted in July 2019, with fifth year (*n* = 114; 79 males and 35 females) and sixth year (*n* = 121; 81 males and 40 females) students attending all the stations separately on the same day. There was no significant difference in the ratio of genders between the two cohorts, and the male/female ratio was comparable to the general demographics of this medical school (1.91). To prevent cheating on the examination, all students were prohibited from using smartphones and other digital devices. Meeting times and locations of each cohort were separated to prevent them from contacting each other.

All the examinees agreed to analyze the quantitative data. In the quantitative analysis, there were no missing data for any item. The scores assessed with three different scales are shown in Table 3. The total IRS scores of the fifth and sixth years cohorts were 52.64 ± 5.08 standard deviation (*SD*) and 59.03 ± 5.27 *SD*, which showed statistically significant differences (*p* < 0.01). The IRS in each station also showed a statistically significant difference. The total TLRS was 3.22 ± 0.42 *SD* vs. 3.94 ± 0.39 *SD* (*p* < 0.01), which was equivalent to year-level mastery. In contrast, the yGRS showed no significant difference between academic year cohorts. Effect sizes *(d)* of IRS and TLRS were medium to large, while yGRS showed negligible effect sizes.

**Table 3:** Comparison of examination results between the year cohorts. IRS and TLRS showed a statistically significant difference between the cohorts, while yGRS did not

	scales	5th (*n* = 114)		6th (*n* = 117)		*F*	*p*	*d*
		mean	*SD*	mean	*SD*			
Clinical procedure (Suturing)	IRS	16.13	2.83	18.56	3.33	1.28	< 0.01	0.79
	TLRS	2.99	0.70	3.62	0.82	15.26	< 0.01	0.82
	yGRS	4.13	0.77	4.03	1.00	7.62	0.37	0.12
Patient safety (Surgical marking)	IRS	16.72	2.75	19.31	2.47	1.44	< 0.01	0.99
	TLRS	3.23	0.58	4.03	0.52	7.93	< 0.01	1.46
	yGRS	4.26	0.61	4.31	0.71	4.26	0.61	0.07
Patient note	IRS	19.79	2.67	21.15	2.03	3.86	< 0.01	0.58
	TLRS	3.45	0.80	4.17	0.46	53.23	< 0.01	1.11
	yGRS	4.55	0.67	4.41	0.63	0.39	0.10	0.22
Total	IRS	52.64	5.08	59.03	5.27	0.16	< 0.01	1.23
	TLRS	3.22	0.42	3.94	0.39	2.71	< 0.01	1.78
	yGRS	4.32	0.44	4.25	0.52	4.52	0.28	0.14

*IRS* item rating scale, *SD* standard deviation, *TLRS* training level rating scale, *yGRS* year-adjusted rating scale

The correlations between the scales across the stations were large, as shown in Table 4. Inter-station reliabilities (alpha) were calculated as 0.65 for the IRS, 0.58 for the yGRS and 0.80 for the TLRS.

**Table 4:** Correlations between the scales. The large strength of association between the scales was observed in all stations

		*r*_s_	*p*
Clinical procedure	IRS - TLRS	0.79	< 0.01
	IRS - yGRS	0.59	< 0.01
	TLRS - yGRS	0.68	< 0.01
Patient safety	IRS - TLRS	0.79	< 0.01
	IRS - yGRS	0.60	< 0.01
	TLRS - yGRS	0.54	< 0.01
Patient note	IRS - TLRS	0.77	< 0.01
	IRS - yGRS	0.68	< 0.01
	TLRS - yGRS	0.44	< 0.01
Total	IRS - TLRS	0.85	< 0.01
	IRS - yGRS	0.56	< 0.01
	TLRS - yGRS	0.45	< 0.01

*IRS* item rating scale, *TLRS* training level rating scale, *yGRS* year-adjusted rating scale.

For the qualitative analysis, 68 fifth year students provided anonymous comments regarding the impact of the OSCE, thus yielding 68 quotes. After the open coding, similar codes were grouped into categories and sub-categories. With the exception of 20 codes unrelated to the research questions (e.g., claims for logistical management of the examination), thematic saturation was reached after analyzing the data. Then we divided categories in view of the promoters and inhibitors of the SDL framework (self-management, motivation, and self-monitoring) [12].

Four categories were identified for the SDL framework of this study: students indicated (1) “promoted motivation to learn during the clinical clerkship,” in addition to experiencing (2) “dissatisfaction with being asked about tasks they have not experienced.” Furthermore, (3) “confusion about not being able to use previous test-cracking strategies” was observed, due to (4) an “unfamiliarity to competencies at graduation.”

The subcategories *Satisfaction with assessment on training results*, *Willingness to have more clinical experience in clerkship*, and *Seeking feedback* were classified under the first category *Promoted motivation to learn during the clinical clerkship*.

The subcategories, *Realizing unexperienced procedures and materials, Confronting with scarce skill training*, and *Notice of clinician educator’s wrong procedure* were aggregated into the second category *Dissatisfaction with being asked about tasks they have not experienced.*

The subcategories *Complaints about that no specific coverage was shown beforehand, Taking wrong test-cracking strategies,* and *Focused only on knowledge domain* was classified under the third category *Confusion about not being able to use previous test-cracking strategies.*

Finally, the subcategories *Insufficient understanding of the required task at graduation* and *Insufficient understanding of the required level at graduation* were created under the category, *Unfamiliarity to competencies at graduation.* Table 5 shows the categories, subcategories and representative quotes.

**Table 5:** Frequency of codes for each category and subcategory. Four categories were identified for the self-directed learning framework [16] of this study

Categories	Subcategories	Typical quotes	Self- motivation	Self- monitoring	Self- management
Promoted motivation to learn during the clinical clerkship (21)	Satisfaction with assessment on training results (10)	*“I thought I showed enough performance in the well-simulated situation.” (#48)* *“The stations were something that could be applied to what I experienced, rather than general knowledge.” (#51)*	promoter	promoter	
	Willingness to have more clinical experience in clerkship (10)	“*I felt the actual workplace would be more diverse and complex.” (#15)* *“I felt pressured because I was not able to do enough tasks that I should have been able to do upon graduation. I’m glad I got to experience it now.” (#49)*			
	Seeking feedback (1)	*“It would have been even better for learning to get feedback immediately.” (#12)*			
Dissatisfaction with being asked about tasks they have not experienced (16)	Realizing unexperienced procedures and materials (8)	*“I have never used suture needles with thread, although seen them.” (36)* *“I have difficulty understanding what the stations were.” (#43)*		promoter	inhibitor
	Confronting with scarce skill training (6)	*“I have had little experience on such tasks.” (36)*			
	Notice of clinician educator’s wrong procedure (2)	*“I realized what my attending physician taught me was not correct after the exam”. (#1)*			
Confusion about not being able to use previous test-cracking strategies (6)	Complaints about that no specific coverage was shown beforehand (4)	*“There is no idea what I should study for the exam.” (#12)*			inhibitor
	Taking wrong test-cracking strategies (2)	*“The textbook told me general procedures, but it was insufficient when I was asked what to do in the workplace.” (#45)*			
	Focused only on knowledge domain (1)	*“I spent a lot of time reviewing clinical reasoning, but not enough on skills.” (#7)*			
Unfamiliarity to competencies at graduation (5)	Insufficient understanding of the required task at graduation (3)	*“This task is unfair because some students experienced while others not at this point.” (#44)*		inhibitor	
	Insufficient understanding of the required level at graduation (2)	*“It was too difficult for the fifth-year students to take the exam.” (#4)*			

## Discussion

PT has been used in some medical schools, primarily in Europe, to improve knowledge-based competencies [1]. Although PT is generally resource-intensive [12], the concept is becoming more widespread as competency-based curricula in medical education have become more widely known. The use of written examinations has been shown to be beneficial in providing feedback to trainees and educators, monitoring growth over time, and promoting deeper learning by shifting the focus from assessment *of* learning to assessment *for* learning [20]. However, the usefulness of written examinations is limited when assessing many of the skills required from physicians, such as physical examinations and the ability to communicate with patients. Although OSCEs are expensive to administer, they are a viable means of assessing clinical skills [21], and their predictive validity for future performance has been studied [22, 23].

As demonstrated in this study, OSCEs in undergraduate education can be used as PT. OSCE was found to be a tool for observing the progression of competencies, showing higher scores and effect sizes for more experienced students. yGRS showed negligible effect sizes and high reliability, which means this scale can be used for summative assessment within each cohort. The analysis of individual stations showed that the mean test scores from all three rating scales had high inter-rater reliability. The scores on each rating scale were highly correlated. The IRS and TLRS scores differed between the training levels at all stations, suggesting that they were good at identifying levels of expertise across all content areas. yGRS scores, on the other hand, are useful for making complex multifaceted judgments, such as summative assessments. As each measure serves a different purpose, it would be beneficial to include all of them.

Therefore, the TLRS could be used to provide individual feedback to students, showing their overall ranking and the IRS could be used to communicate the level of achievement of individual items. By contrast, the yGRS could be used for a summative assessment of test results. These characteristics are similar to those in studies conducted on postgraduate education [4, 11]. These studies were conducted on residents in their first to fourth year of residency, whereas ours was conducted on students from two clinical years and therefore showed two levels of progress. Although shorter, we were able to measure progress in competencies similar to previous studies, suggesting that OSCE-PT is useful in undergraduate clinical education. A potential avenue for OSCE-PT is a scale to gauge entrustability, enabling the generation of reliable assessments capable of differentiating learners across diverse training levels in the postgraduate setting [11]. In this study, we designated “entrusted to perform” as the highest level in the TLRS; however, it was not deemed indicative of measuring the entrustability level or milestones. While the assessment of entrustability inclinical procedures remains limited for undergraduate students, there exists an opportunity to explore the applicability of OSCE-PT in assessing clinical activities permitted for students.

The impact of rating stringency presents another challenge in OSCE-PT, and it is known that the impact of differences in rating stringency differs between procedural and non-procedural tasks [24]. While our OSCE included two procedural tasks (clinical procedure, patient safety procedure) and one non-procedural task (patient note), we found no difference. Further research is needed to determine the level of stringency that best motivates students within an environment accustomed to high-stakes examinations.

The results of the qualitative analysis showed that SDL can be facilitative, especially in terms of motivation. However, the results also suggest that it could inhibit self-management and self-monitoring. Much of this was owing to the fact that the curriculum and educational opportunities were not sufficiently aligned with the current testing opportunity. The original PT established its significance on a vertically integrated curriculum [25]. Students in this curriculum can be aware of their final competencies from the earliest stages and continue to engage in their learning. A trial of PT in a non-integrated curriculum revealed that the increased motivation of students through examinations did not necessarily lead to the establishment of self-directedness 26]. These results are consistent with those in the present study. Furthermore, our attempt is about performance training, which requires more readiness for learning than knowledge acquisition because it is more difficult to start learning independently.

Additionally, aspects of self-management and monitoring as well as competencies at the end of the clinical program are difficult to convey to students, despite the fact that these competencies were explained to them from the time they enrolled in the clinical clerkship. One of the characteristics that distinguishes the undergraduate clinical clerkship from postgraduate training is that it rotates through many departments, and the students are less independent in the undergraduate curriculum than in the residency. Strategies for encouraging consistent monitoring, management, and support training were considered to be inadequate. Planned opportunities must be incorporated into the clinical clerkship curriculum to enable students to train more thoroughly and regularly. Providing extra-curricular skill training is also helpful for motivated students. When introducing the OSCE-PT to undergraduates, a coordinated effort with ongoing support across the curriculum, and not only for the exam, was considered necessary. Especially, the opportunities for learning after the examination, like post-test training, will engage students more in learning [26].

Notably, we were able to measure progress in competencies as PTs in the issue of patient safety. Since the publication of the Patient Safety Curriculum Guide by the World Health Organization [27], patient safety education has received increasing attention in recent years. Education is necessary not only for post-accident measures but also for quality improvement; however, there are areas where the undergraduate curriculum does not adequately meet needs, such as diagnostic errors [28] and medication safety [29]. In addition, it is difficult to incorporate appropriate methods for assessing competencies into the curriculum. Self-reflection and portfolio are often used but sometimes susceptible to social desirability bias in issues where the ideal response is evident [30], such as patient safety. Also, in workplace-based assessments, consistency is a concern when dealing with patient safety events that are highly context-dependent. By contrast, OSCE is suitable for the summative assessment of patient safety because it can assess competencies collectively in a validity-controlled context. In the future, OSCE tasks for patient safety should be developed, and further utilization should be explored to use them as a PT.

### Limitations

This study had several limitations. First, the generalizability of results is constrained by the single institution setting and a comparison involving a small number of OSCE stations. Additionally, we did not compare PT with written test performance, despite a prior study at a Japanese medical university suggesting potential benefits of PT in the knowledge domain [26]. Further studies with more stations in multiple schools will be warranted.

Second, there were concerns regarding the reliability of the examiners. Although they had experience with existing OSCE assessments, they were not blinded to the examinees’ academic years, which may have anchored them to the differences in ability. However, given the correlation between the ratings and the actual academic years, we can conclude that the examiners were able to assess the students independently based on their actual academic years. In addition, there was one rater at each station. Therefore, we could not test for inter-rater errors or other rater characteristics. As a pilot study, we assigned one rater at each station to maintain feasibility; if the OSCE-PT is conducted as a higher-stakes examination, it would be more reliable to assess it with multiple raters. However, OSCE generally requires significant resources, and so does PT [20]. The utility of an assessment method is not only determined by reliability, but also educational impact, acceptability and cost [31]. If educational impact through feedback (i.e., formative assessment) is the primary purpose, as in the original PT, the number of raters should be determined by overall utility.

Third, this pilot study was conducted before the onset of the COVID-19 pandemic. After the pandemic, the situation regarding the OSCE changed slightly, and there has been a shift back to a workplace-based assessment [32]. However, workplace-based assessments have not been fully implemented yet [33]. In East Asian cultures, where students are aware of exceptionally reliable examinations, both faculty and students must establish assessments using standardized tasks rather than case-specific workplace assessments [34]. As it has been reported that even summative assessments can be effective in promoting learning if they are highly authentic, it is expected that the OSCE-PT will promote the learning of performance competencies.

## Conclusions

This study provides a rationale for using the OSCE-PT. IRS scores and TLRS as indicators of progress and the yGRS as a summative assessment for each year. Student responses were consistent with previous studies on the knowledge assessment of PTs in Japan. More detailed information on the purpose of PTs and the use of feedback should be provided.

### Supplementary Information

**Additional file 1.**

## Data Availability

The datasets generated and analyzed during the current study are not publicly available due to the participants’ data and anonymity but are available from the corresponding author upon written request.

## Abbreviation

CAT: Common Achievement Tests
CC: Clinical Clerkship
IRS: Item Rating Scale
OSCE: Objective structured clinical examination
OSCE-PT: Objective structured clinical examination as a progress testing
PT: Progress testing
SDL: Self-directed learning
TLRS: Training Level Rating Scale
yGRS: year-adjusted Global Rating Scale
