# Supplementary material for: Progress testing of an objective structured clinical examination during undergraduate clinical clerkship: a mixed-methods pilot study
Source: BMC Med Educ. 2023 Dec 14;23:958. doi: 10.1186/s12909-023-04940-8 (PMC10720173; doi:10.1186/s12909-023-04940-8)
Supplement: Supplementary file 1 — Additional file 1. [file 12909_2023_4940_MOESM1_ESM.docx]

|  | | | |  | | | |  | | | | | |  | | | | | | |  |
| --- | --- | --- | --- | --- | --- | --- | --- | --- | --- | --- | --- | --- | --- | --- | --- | --- | --- | --- | --- | --- | --- |
| OSCE rating sheet (example)  Redacted | | | | | | Station  No. | | | ID | | | NAME | | | | | | | | |  |
| Item Rating Scale | | | | | | | | | | | | Same as TLRS | | | | | | | | |  |
| A | |  | | | | | | | | | | ⓐ | | | ⓑ | ⓒ | ⓓ | | ⓔ | ⓕ |  |
| B | |  | | | | | | | | | | ⓐ | | | ⓑ | ⓒ | ⓓ | | ⓔ | ⓕ |  |
| C | |  | | | | | | | | | | ⓐ | | | ⓑ | ⓒ | ⓓ | | ⓔ | ⓕ |  |
|  | |  | | | | | | | | | |  | | |  |  |  | |  |  |  |
|  | |  | | | | | | | | | |  | | |  |  |  | |  |  |  |
| Training Level  Rating Scale | | | Preclinical  Not allowed to start CC | | Just starting CC | | during CC | | | Completing CC  Acceptable to graduate | | | During residency | | | | | Completing residency  Can be entrusted to perform | | |  |
|  |  |  | ⓐ | | ⓑ | | ⓒ | | | ⓓ | | | ⓔ | | | | | ⓕ | | |  |
|  | | |  | |  | |  | | |  | | |  | | | | |  | | |  |
| Global Rating Scale  (Year-adjusted) | | | Inferior | | Poor | | Borderline | | | Acceptable | | | Good | | | | | Excellent | | |  |
|  |  |  | ① | | ② | | ③ | | | ④ | | | ⑤ | | | | | ⑥ | | |  |
|  |  | |  | |  | |  | | |  |  | |  | | | | |  | | |  |
|  |  | |  | |  |  |  | | |  | Name of Rater | | | | | | |  | | |  |
|  |  | |  | |  | |  | | |  |  | |  | | | | |  | | |  |
|  |  | |  | |  | |  | | |  |  | |  | | | | |  | | |  |
|  |  | |  | |  | |  | | |  |  | |  | | | | |  | | |  |
|  |  | |  | |  | |  | | |  |  | |  | | | | |  | | |  |
